# Nutrient patterns and non-alcoholic fatty liver disease in Iranian Adul: A case-control study

**DOI:** 10.3389/fnut.2022.977403

**Published:** 2022-09-06

**Authors:** Ammar Salehi-sahlabadi, Farshad Teymoori, Hamid Ahmadirad, Ebrahim Mokhtari, Mina Azadi, Shaikh Sanjid Seraj, Azita Hekmatdoost

**Affiliations:** ^1^Student Research Committee, Department of Clinical Nutrition and Dietetics, Faculty of Nutrition and Food Technology, National Nutrition and Food Technology, Shahid Beheshti University of Medical Sciences, Tehran, Iran; ^2^Nutrition and Endocrine Research Center, Research Institute for Endocrine Science, Shahid Beheshti University of Medical Sciences, Tehran, Iran; ^3^Department of Nutrition, School of Public Health, Iran University of Medical Sciences, Tehran, Iran; ^4^Walsall Healthcare NHS Trust, Walsall Manor Hospital, West Midlands, Walsall, United Kingdom; ^5^Department of Clinical Nutrition and Dietetics, Faculty of Nutrition and Food Technology, National Nutrition and Food Technology, Research Institute, Shahid Beheshti University of Medical Sciences, Tehran, Iran

**Keywords:** nutrient patterns, non-alcoholic fatty liver disease, NAFLD, pattern, diet

## Abstract

**Backgrounds:**

The current literature boasts the importance of diet in preventing or managing liver complications. However, there is limited evidence on the association of nutrient patterns (NP) with these complications. In this case-control study, we aimed to examine the possible relationship between nutrient patterns and the risk of non-alcoholic fatty liver disease (NAFLD) amongst the adult Iranian population.

**Methods:**

This case-control study is being conducted at the Metabolic Liver Disease Research Center at Isfahan University of Medical Sciences in 2019. The study included 225 newly diagnosed cases of NAFLD and 450 controls. A validated semi-quantitative food frequency questionnaire (FFQ) assessed dietary intake. Principal component analysis using Varimax rotation obtained nutrient patterns. Logistic regression was performed to estimate NAFLD risk.

**Results:**

We identified four major nutrient patterns. The first nutrient pattern was high in consumption of lactose, animal protein, vitamin D, riboflavin, pantothenic acid, vitamin B12, calcium, phosphorus, zinc, and potassium. The second nutrient pattern included fiber, plant protein, vitamin A, thiamine, niacin, copper, and selenium, while the third featured plant protein, zinc, copper, magnesium, manganese, chromium, and selenium. The fourth was characterized by fructose, vitamin A, pyridoxine, vitamin C, and potassium. After adjusting for confounders, individuals in the highest tertile of NP4 had lower odds of NAFLD (OR: 0.56, 95% CI: 0.32–0.98, *P*_trend = 0.042); compared to those who were in the lowest tertile.

**Conclusion:**

High compliance to a nutrient pattern characterized by fructose, vitamin C, vitamin A, pyridoxine, and potassium mainly supplied from fruits, vegetables, and nuts is inversely proportional to the odds of NAFLD. Also our findings indicate a very high fiber intake, a relatively optimal dietary fat profile, and a pretty low sugar intake for cases and controls, unseen in western countries. However, these initial findings need to be approved with further studies to confirm the relationship between nutrient patterns and NAFLD.

## Background

Non-alcoholic fatty liver disease (NAFLD) is a progressive chronic condition that ranges from hepatic steatosis to fibrosis and end-stage cirrhosis. NAFLD is the accumulation of more than 5% fat in the liver not caused by alcohol consumption or hepatic viral infections (1). NAFLD is associated with other metabolic-related disorders such as obesity, insulin resistance (IR), type 2 diabetes mellitus (DMT2), hypertension, metabolic syndrome (MetS), and hyperlipidemia (2–5). NAFLD has an estimated global adult prevalence of 25%, with the Middle East at 32% (6). Diet is one of the determining factors in both progression and prevention of liver disease (1).

The relationship between diet and NAFLD is investigated broadly at varying levels, including dietary patterns (DP), individual foods, or nutrients pattern (NP) (7–11). The study of individual nutrients has helped to identify deficiencies and diseases. It is suggested that chronic diseases may be influenced by a combination of nutrients (12). Recently, dietary pattern analysis has emerged in nutritional epidemiology that examines the relationship between diet and disease. In this approach, statistical methods combine several foods or nutrients to extract dietary or nutrient patterns (NP). It has been suggested that such patterns may provide a more inclusive insight into diet and disease relationships. In addition, the effect of individual nutrients or foods may be too small to detect, while a combination of them within a pattern can be more predictive of chronic disease (13).

Two studies investigated the link between nutrient patterns and the risk of NAFLD. Tien et al. (14) extracted a nutrient pattern characterized by; folate, carotene, insoluble dietary fiber, vitamin C, iron, retinol equivalent, soluble dietary fiber, potassium, vitamin E, and vitamin B2, which was inversely related to NAFLD risk. Mazidi et al. (15) derived two nutrient patterns. The first one included high fat and carbohydrates, and the second included minerals, vitamins, and fiber, decreasing the risk of hepatic steatosis. It has been demonstrated that higher adherence to an NP rich in vitamin A, vitamin C, pyridoxine, potassium, and fructose is inversely proportional to the three-year changes in fasting serum insulin concentration and HOMA-IR level that may reduce the risk of liver disease (12).

This study aimed to examine the possible relationship between nutrient patterns and the risk of NAFLD amongst the adult Iranian population.

## Methods

### Study population

This case-control study was conducted in the Metabolic Liver Disease Research Center, affiliated with Isfahan University of Medical Sciences. Participants were obtained through convenience sampling. In total, 675 participants aged between 20 and 60 were recruited for this study. Of those, 225 were newly diagnosed NAFLD patients and 450 controls. The details of the study were previously reported (12). In addition to the absence of alcohol consumption and other liver disease causes, an ultrasonography scan of the liver confirmed a NAFLD diagnosis (Grade II, III as an indication of a definite diagnosis). Among other healthy individuals, a control group was selected after liver ultrasonography (without hepatic steatosis stages). For this study, inclusion criteria were: (1) not being on a special diet, (2) not having overt kidney or liver disease (Wilson's disease, autoimmune liver disease, virus infection, and alcoholic fatty liver), (3) not having cardiovascular disease, severe gastrointestinal disease, malignancy, thyroid disorder, and autoimmune disease. (4) Individuals who used drugs that could be hepatotoxic or steatogenic were not included in the study. Participants who completed fewer than 35 food frequency questionnaire items and reported under- or over-reported daily energy intake were excluded (800 kcal/day or 4,500 kcal/day).

### Dietary assessment

Participants' dietary intakes were collected using a valid and reliable 168-item semi-quantitative food frequency questionnaire (FFQ) (16). The FFQ listed a set of common Iranian foods with standard serving sizes. Participants were asked to express their mean dietary intake during the previous year by choosing one of the following categories: never or less than once a month, 3–4 times per month, once a week, 2–4 times per week, 5–6 times per week, once daily, 2–3 times per day, 4–5 times per day, and 6 or more times a day. Portion sizes of each food item were converted into grams using standard Iranian household measures (17). Daily energy and nutrient intake for each individual were calculated using the United States Department of Agriculture's (USDA) Food Composition Table (FCT) (18). The Iranian FCT was used for some traditional foods that do not exist in USDA FCT (19). Then the consumed foods frequencies were transformed into a daily intake scale (12).

### Assessment of other variables

In order to calculate the SES score, three variables were taken into consideration: family size (≤4, >4 people), education (academic or not), and house ownership or not. Depending on whether members of the family were ≤4, academically educated, or owned a home, participants received a score of 1 or a score of 0 (if they were >4, or had no academic education, or leased property). In order to calculate the total SES score, the assigned scores were summed up (minimum SES score of 0 - maximum SES score of 3). High, moderate, and low SES were defined as participants with scores of 3, 2, and sum of subjects with 1 and o, respectively. Participants were interviewed face-to-face to measure their physical activity using the International Physical Activity Questionnaire (IPAQ). We expressed all IPAQ results in Metabolic Equivalents per Week (METs/week).

### Statistical analysis

Statistical analysis was performed using Statistical Package Software for Social Science, version 21 (SPSS Inc., Chicago, IL, United States). Data normality was tested using Kolmogorov-Smirnov's test and histogram chart. The characteristics and dietary intakes of cases and controls were expressed as mean ± SD or median (25–75 interquartile range) and frequency (percentages). The data were compared between cases and controls using an independent sample *t*-test and chi-square for continuous and categorical variables, respectively.

Factor analyses were conducted using the 34 nutrients, including sucrose, lactose, fructose, fiber, animal protein, plant protein, saturated fatty acids, monounsaturated fatty acids, polyunsaturated fatty acids, cholesterol, vitamins A, D, E, K, C, thiamine (B1), riboflavin (B2), niacin (B3), pantothenic acid (B5), pyridoxine (B6), folate (B9), B12, calcium, phosphorus, iron, zinc, copper, magnesium, manganese, chromium, selenium, sodium, potassium, and caffeine. The dietary intake of 34 nutrients per 1,000 Kcal energy intake was calculated and used as input variables. NPs were derived from principal component analysis (PCA) with varimax rotation based on the correlation matrix. Factor scores for all participants for each extracted factor were calculated by summing the frequency of consumption and then multiplied by factor loadings across all 34 nutrients. Statistical correlation between variables and adequacy of sample size was tested using the Bartlett test of sphericity (*P* < 0.001) and the Kaiser-Mayer-Olkin test (0.71), respectively. Four dominant factors were identified based on the scree plot (eigenvalue > 2).

The correlation coefficient between NPs and food sources adjusted for age, sex, and energy intake was calculated using the partial correlation coefficient test. The association between tertiles of NPs and odds of NAFLD was assessed using a logistic regression test adjusted for potential confounding variables, including age, sex, BMI, physical activity, smoking, SES, and dietary intake of energy. The odds ratio (OR) with a 95% confidence interval (CI) of NAFLD across tertiles of NPs was reported. *P*-values < 0.05 were considered statistically significant.

## Results

The mean (SD) age and BMI of participants (53% male) were 38.1 (8.8) years and 26.8 (4.3) kg/m2, respectively. Using the factor analysis on the dietary intakes of 34 nutrients, four main NPs with eigenvalues >2 were identified. Table 1 shows the factor loadings of four major NPs, which explained 59.5% of the total variation of nutrients. The first pattern was characterized by high intakes of lactose, animal protein, vitamin D, riboflavin, pantothenic acid, vitamin B12, calcium, phosphorus, zinc, and potassium. The second pattern was positively correlated with fiber, plant protein, vitamin A, thiamine, niacin, copper, and selenium. This pattern negatively correlated with sucrose, saturated, and monounsaturated fatty acids. The third pattern was high intakes of plant protein, zinc, copper, magnesium, manganese, chromium, and selenium. The fourth pattern was characterized by a high intake of fructose, vitamin A, pyridoxine, vitamin C, and potassium.

**Table 1:** Factor loading matrix and explained variances for major nutrient patterns identified by factor analysis in 225 cases and 450 controls ^*^^†^.

	**Nutrient patterns**			
**Nutrients**	**Pattern 1**	**Pattern 2**	**Pattern 3**	**Pattern 4**
Sucrose		**-0.40**		
Lactose	**0.91**			
Fructose				**0.85**
Fiber		**0.76**		
Animal protein	**0.67**			
Plant protein		**0.68**	**0.53**	
Saturated fatty acids	0.36	**-0.48**		
Mono unsaturated fatty acids		**-0.43**		
Poly unsaturated fatty acids	−0.30			
Cholesterol				
Vitamin A		**0.78**		**0.49**
Vitamin D	**0.63**			
Vitamin E				
Vitamin K				
Thiamine		**0.74**		**-0.32**
Riboflavine	**0.88**			
Niacin		**0.50**		
Pantothenic acid	**0.76**			
Pyridoxine				**0.46**
Folate				
Vitamin B12	**0.49**			
Vitamin C				**0.85**
Calcium	**0.87**			
Phosphor	**0.86**		0.32	
Iron				
Zinc	**0.58**		**0.49**	0.35
Copper		**0.64**	**0.42**	0.31
Magnesium	0.38		**0.75**	
Manganese			**0.71**	
Chromium			**0.94**	
Selenium		**0.41**	**0.77**	−0.30
Sodium				
Potassium	**0.50**			0.64
Caffeine				
**Explained variance (%)**	22.30	19.95	9.67	7.56
**Cumulative explained variance (%)**	22.30	42.25	51.93	59.5

* Principal Component Analysis (PCA) performed on 34 nutrients calculated as intake per 1,000 Kcal.

Nutrients with loadings >0.40 and < −0.40 (in bold) are being characteristic for the four patterns; loadings < 0.3 (in absolute value) are suppressed.

† Kaiser's Measure of Sampling Adequacy, KMO = 0.71, Bartlett's test of sphericity = <0.001.

Table 2 presents the general characteristics and dietary intakes of cases and controls. NAFLD patients with higher BMI and SES scores were more smokers and had lower physical activity than the control group (*p* < 0.05). Also, NAFLD patients had a higher dietary intake of energy, plant protein, and thiamine. However, have lower intakes of sucrose, vitamin A, and vitamin D (*p* < 0.05). The two study groups had no significant difference in other variables (*p* > 0.05).

**Table 2:** Characteristics and dietary intakes among cases and controls^*^.

**Demographic variables**	**Cases (*n* = 225)**	**Controls(*n* = 450)**	***P*-value**
Age(year)	38.6 ± 8.7	37.8 ± 8.9	0.293
Male, *n* (%)	125 (55.6)	233 (51.8)	0.354
BMI(Kg/m^2^)	30.6 ± 4.0	25.0 ± 3.1	<0.001^a^
Smoking, *n* (%)	16 (7.1)	12 (2.7)	0.008^a^
Physical activity (MET/min/week)	1119 ± 616	1590 ± 949	<0.001^a^
**SES**, ***n*** **(%)**			0.022^a^
Low	65 (28.9)	158 (35.1)	
Middle	104 (46.2)	206 (45.8)	
High	56 (24.9)	86 (19.1)	
**Nutrient intake**			
Energy intake (Kcal/d)	2369 ± 621	2227 ± 645	0.006^a^
Sucrose(g/1,000 Kcal)	12.9 ± 6.4	14.3 ± 8.6	0.012^a^
Lactose(g/1,000 Kcal)	6.8 ± 4.2	7.4 ± 4.5	0.126
Fructose(g/1,000 Kcal)	7.8 ± 3.3	7.9 ± 3.6	0.662
Fiber(g/1,000 Kcal)	16.8 ± 8.3	15.9 ± 6.5	0.154
Animal protein(g/1,000 Kcal)	22.8 ± 8.6	21.6 ± 8.5	0.072
Plant protein(g/1,000 Kcal)	14.5 ± 3.7	13.8 ± 3.4	0.024^a^
Saturated fatty acids(g/1,000 Kcal)	11.4 ± 3.4	11.7 ± 3.2	0.269
Mono unsaturated fatty acids(g/1,000 Kcal)	12.0 ± 3.3	11.8 ± 2.9	0.632
Poly unsaturated fatty acids(g/1,000 Kcal)	7.3 ± 2.8	7.1 ± 2.4	0.211
cholesterol(mg/1,000 Kcal)	94.4 ± 41.4	101.7 ± 56.0	0.057
Vitamin A(mg/1,000 Kcal)	191 ± 112	216 ± 112	0.008^a^
Vitamin D(μg/1,000 Kcal)	0.72 ± 0.55	0.93 ± 0.74	<0.001^a^
Vitamin E(mg/1,000 Kcal)	5.01 ± 1.53	5.03 ± 1.74	0.859
Vitamin K(mg/1,000 Kcal)	78.0 ± 52.3	82.9 ± 57.2	0.267
Thiamine(mg/1,000 Kcal)	0.86 ± 0.18	0.82 ± 0.16	0.007^a^
Riboflavine(mg/1,000 Kcal)	0.84 ± 0.22	0.87 ± 0.20	0.110
Niacin(mg /1,000 Kcal)	9.7 ± 2.3	9.5 ± 2.0	0.331
Pantothenic acid(mg/1,000 Kcal)	2.33 ± 0.42	2.38 ± 0.44	0.151
Pyridoxine(mg/1,000 Kcal)	0.82 ± 0.16	0.82 ± 0.15	0.692
Folate(mg/1,000 Kcal)	231 ± 45	228 ± 38	0.526
Vitamin B12(mg/1,000 Kcal)	1.72 ± 0.86	1.78 ± 0.79	0.422
Vitamin C(mg/1,000 Kcal)	58.7 ± 31.1	60.4 ± 31.5	0.509
Calcium(mg/1,000 Kcal)	528 ± 163	532 ± 152	0.723
Phosphor(mg/1.000 Kcal)	621 ± 127	622 ± 119	0.923
Iron(mg/1,000 Kcal)	11.6 ± 5.4	11.7 ± 5.7	0.881
Zinc(mg/1,000 Kcal)	4.88 ± 0.89	4.85 ± 0.85	0.689
Copper(mg/1,000 Kcal)	0.64 ± 0.13	0.6439 ± 0.13073	0.896
Magnesium(mg/1,000 Kcal)	158 ± 29	159 ± 30	0.789
Manganese(mg/1,000 Kcal)	3.1 ± 1.1	3.1 ± 1.0	0.951
Chromium(mg/1,000 Kcal)	0.03 (0.02–0.05)	0.03 (0.01–0.05)	0.486
Selenium(mg/1,000 Kcal)	48.0 ± 12.2	47.5 ± 11.7	0.588
Sodium(mg/1,000 Kcal)	1990 ± 1960	2040 ± 1385	0.736
Potassium(mg/1,000 Kcal)	1546 ± 365	1596 ± 372	0.093
Caffeine(mg/1,000 Kcal)	55.4 ± 46.0	59.1 ± 44.8	0.316

* Data are presented as mean ± standard deviation or median (IQR) for continues variables, and number and percentages for categorical variables.

The differences of continues and categorical variables between cases and controls were assessed using independent sample t-test and chi square were ^a^p < 0.05.

The partial correlation coefficient of NPs with food sources is presented in Table 3. NP1 showed a strong significant positive correlation with low-fat dairy, a weak positive correlation with high-fat dairy, egg, and plant oil, and a weak negative correlation with refined grains, artificial beverages, and snacks. NP2 indicates a moderate positive correlation with refined grain, a weak positive with legumes and whole grain, and a weak negative correlation with low-fat dairy, high-fat dairy, nuts, fruit juice, snacks, artificial beverages, and plant oil. We observed a strong positive correlation with whole grains in NP3. However, nuts had weak positive, and refined grains, high-fat dairy, fruits, artificial beverages, and snacks showed weak negative correlations with this NP. The fourth NP respectively showed a strong positive correlation with fruits weak and moderate positive correlation with vegetables, fruit juice, nuts, artificial beverages, and plant oil. In contrast, this pattern showed a moderate and weak negative correlation with refined grains, red-processed meats, high-fat dairy, eggs, and snacks.

**Table 3:** Partial correlation coefficient of nutrient patterns with food sources^*^.

	**Nutrient pattern 1**	**Nutrient pattern 2**	**Nutrient pattern 3**	**Nutrient pattern 4**
Whole grain(g/d)	−0.026	0.141^a^	0.752^a^	−0.087^b^
Refined grain(g/d)	−0.239^a^	0.427^a^	−0.194^a^	−0.360^a^
Legume(g/d)	−0.006	0.155^a^	0.071	0.008
Nuts(g/d)	−0.012	−0.152^a^	0.131^b^	0.216^a^
Red and processed meat(g/d)	−0.017	−0.093^b^	−0.011	−0.105^b^
White meats(g/d)	−0.035	−0.052	−0.007	0.07
Low fat dairy(g/d)	0.705^a^	−0.172^a^	−0.099^b^	0.046
High fat dairy(g/d)	0.230^a^	−0.113^b^	−0.113^b^	−0.123^b^
Fruits(g/d)	0.047	−0.073	−0.115^b^	0.810^a^
Vegetables(g/d)	0.097^b^	0.035	−0.051	0.369^a^
Egg (serving/d)	0.091^b^	0.034	0.002	−0.091^b^
Fruit juice (serving/d)	0.047	−0.082^b^	−0.034	0.385^a^
Snacks (serving/d)	−0.129^b^	−0.300^a^	−0.120^b^	−0.080^b^
Artificial bavarages (serving/d)	−0.111^b^	−0.086^b^	−0.092^b^	0.085^b^
Plant oil (serving/d)	0.095^b^	−0.014	0.047	0.101^b^

* Adjusted for age, sex, and energy intake.

a P < 0.001,

b P < 0.05.

The associations between NPs and NAFLD are demonstrated in Table 4. In the age and sex-adjusted model, there was no significant relationship between each NP and NAFLD. after additional adjustment for potential confounders including BMI, physical activity, smoking, SES, and energy intake, participants who were in the highest vs. lowest tertile of NP1 (OR: 0.77, 95% CI: 0.45–1.34, *P*_trend = 0.361), NP2 (OR: 1.04, 95% CI: 0.60–1.81, *P*_trend = 0.780), and NP3 (OR: 1.17, 95% CI: 0.67–2.04, *P*_trend = 0.649) showed no significant association with NAFLD. However, individuals in the highest tertile of NP4 had lower odds of NAFLD (OR:0.56, 95% CI: 0.32–0.98, *P*_trend = 0.042); compared to those who were in the lowest tertile.

**Table 4:** Odds ratios (ORs) and 95% confidence intervals (CIs) for NAFLD based on tertiles of nutrient patterns.

	**Tertiles of nutrient patterns**			***P* for trend**
	**T1**	**T2**	**T3**	
**Nutrient pattern 1**				
Median score	−0.96	−0.12	0.90	
NAFLD /control	78 / 147	78 / 147	69 / 156	
Model 1^*^	1.00 (Ref)	1.00 (0.68–1.47)	0.85 (0.57–1.26)	0.395
Model 2^†^	1.00 (Ref)	0.88 (0.50–1.52)	0.77 (0.45–1.34)	0.361
**Nutrient pattern 2**				
Median score	−0.92	−0.09	0.81	
NAFLD /control	71 / 155	71 / 153	83 / 142	
Model 1^*^	1.00 (Ref)	1.00 (0.67–1.48)	1.24 (0.84–1.84)	0.240
Model 2^†^	1.00 (Ref)	0.88 (0.51–1.53)	1.04 (0.60–1.81)	0.780
**Nutrient pattern 3**				
Median score	−0.85	−0.16	0.80	
NAFLD /control	68 / 157	80 / 145	77 / 148	
Model 1^*^	1.00 (Ref)	1.25 (0.84–1.86)	1.16 (0.78–1.73)	0.529
Model 2^†^	1.00 (Ref)	1.29 (0.74–2.25)	1.17 (0.67–2.04)	0.649
**Nutrient pattern 4**				
Median score	−0.96	−0.13	0.99	
NAFLD /control	76 / 150	83 / 150	66 / 150	
Model 1^*^	1.00 (Ref)	1.08 (0.73–1.59)	0.86 (0.57–1.28)	0.412
Model 2^†^	1.00 (Ref)	0.87 (0.50–1.49)	0.56 (0.32–0.98)	0.042

* Model 1: Adjusted for age and sex.

† Model 2: Additionally adjusted for model 1 and BMI, physical activity, smoking, SES, dietary intake of energy.

## Discussion

In this case-control study, four major NPs were identified. After adjustment for potential confounders, a significant inverse association was observed between the fourth NP, characterized by a high intake of fructose, vitamin A, pyridoxine, vitamin C, and potassium with the odds of NAFLD. We observed no significant association between other NPs and NAFLD.

To our knowledge, two previous cross-sectional studies have examined the relationship between NPs and NAFLD. A study among Japanese adults reported that an NP consisting of vitamin A precursors, Vitamin C, potassium, Vitamin B2, Vitamin E, folate, iron, and fiber could help prevent NAFLD (14). Mazidi et al. (15) indicated that a pattern rich in total fat and carbohydrates such as SFA, MUFA, cholesterol, total sugar, alcohol, and sodium was directly associated with a higher risk of NAFLD. Whereas an NP rich in minerals (calcium, potassium, magnesium, phosphorus, copper), vitamins (B1, B2, B3, folate, vitamins C, A, and E), and fiber were inversely related to the risk of NAFLD.

Iwasaki et al. (20) demonstrated that fiber, potassium, and vitamins pattern and saturated fatty acids, calcium, and vitamin B2 pattern were associated with lowered MetS risks, while fats and fat-soluble vitamins were associated with increased MetS risks. Recently, a study has shown that following a semi-animal nutrient pattern characterized by high intakes of calcium, potassium, vitamins B2, B12, A, D, K, and C, fats, dietary cholesterol, and TFA is positively associated with MetS (21).

Regarding the many differences between studies investigating NPs related to chronic diseases, such as different study designs, populations, outcomes, the number of nutrients included in the factor analysis, etc., NPs have shown much variety, and scarcely an NP has been repeated in studies. Our study is the first case-control study investigating the association between NPs and NAFLD. A study most in line with our results relates to a prospective study that extracted an NP including vitamin A, vitamin C, pyridoxine, potassium, and fructose, and this pattern has been associated with a lower risk of hyperinsulinemia and IR, and dyslipidemia among participants from Tehran (22, 23). Although there are considerable differences between this study and others in TLGS (Tehran lipid and glucose study), such as different study design, sample size, and outcome of the studies, based on the literature reviews, there is a significant correlation between hyperinsulinemia and IR with NAFLD (12). These studies were conducted on Iranian participants, and NPs extracted using similar nutrients. These similar extracted NPs (vitamin A, vitamin C, pyridoxine, potassium, and fructose) and their inverse association with IR, dyslipidemia, and NAFLD, may show the significance and power of this NP among the Iranian population.

The first NP in the present study was a tendency to reduce the odds of NAFLD. However, the distribution of a wide range of nutrients demonstrated both beneficial [potassium (24), vitamin D (25)] and detrimental [animal protein (26)] effects on the fatty liver, leading to the neutralization of pattern strength in displaying a significant association. Furthermore, this NP does not consume much variety of food groups and is mainly limited to dairy and somewhat to fruit and vegetables. We acknowledge that some of the obtained patterns may seem unrealistic, especially NP1, characterized by higher consumption of dairy products and vegetables. However, it is necessary to pay attention to several points in this context; firstly, in the PCA method, different food groups are placed together in a pattern based on the correlation between their nutrients. Secondly, although this food pattern is not dominant in the Iranian population, it is not far from reality in traditional cities like Isfahan. For example, yogurt and doogh, a drink made from yogurt, are consumed with meals. Also, cheese is a popular and widely consumed food in breakfast. Also, different vegetables are consumed both as main food and as side dishes. On the other hand, in Iranian food culture, some foods are prepared only from the combination of dairy products and vegetables.

NPs 2 and 3 showed no relation with NAFLD in our study. Lack of alignment in detrimental nutrients or food groups related to fatty liver in these NPs resulted in any significant association between NPs 2 and 3 with NAFLD. In this study, NP4, despite other NPs, has a positive correlation with beneficial food groups, including fruits, vegetables, and nuts, and a negative correlation with unhealthy food groups such as refined grains, high-fat dairy, and red-processed meat. A prospective cohort study among Korean adults indicated that higher consumption of fruit and vegetables reduced the risk of NAFLD by 23 and 29%, respectively (27). Also, vegetable consumption was inversely related to LDL, TC, and ALT levels in Iranian adults, and fruit consumption was considered to decrease visceral fat positively (28, 29). Several studies showed that higher nut consumption was significantly associated with a lower prevalence of NAFLD (29, 30). These components are an important part of a healthy dietary pattern that lowers the risk of NAFLD (11). In contrast, a Western dietary pattern that consisted mainly of processed meat, high-fat dairy, soft drinks, refined grains, and fast foods was associated with a higher risk of NAFLD (11, 31, 32).

Some mechanisms relate to the beneficial associations between NP 4 components with NAFLD or its risk factors. It is known that vitamins play a role in the pathogenesis of NAFLD in different ways. In non-alcoholic steatohepatitis animal models, vitamin A plays an important role in hepatic fatty acid β oxidation, while vitamin C protects hepatic cells from lipotoxic-induced oxidative stress (33). In a previous study, vitamin C significantly improved liver function and oxidative stress by decreasing mitochondrial ROS and increasing manganese superoxide dismutase and glutathione peroxidase activities (34). Vitamin B6 is recognized as an important cofactor in fat metabolism, and it was observed that B6 administration significantly ameliorates hepatic fat accumulation (35).

Additionally, studies have shown that pyridoxine may improve insulin signaling and prevent endothelial dysfunction and intrahepatic fat accumulation (36). Adequate potassium intake has been shown to prevent obesity and MetS by regulating insulin secretion and carbohydrate metabolism (37). In a recent study, sugar-sweetened beverages appeared to adversely affect peripheral and hepatic insulin sensitivity, even if only in moderate daily amounts. In contrast, fruit-derived fructose did not have any detrimental metabolic effects and showed inverse correlations with the level of hepatocellular fat in individuals with type 2 diabetes (38). Concerning the high correlation of fruits with NP4, it seems that fruits were the main source of fructose. Also, it seems that low levels of refined grains, red-processed meats, and high-fat dairy intake, as observed in NP4, might explain low rates of NAFLD (39–41). A recent cross-sectional study showed that meat, high-fat dairy, pasta, and rice intake were all greater in those with NAFLD (41). The nutritional recommendations for people with fatty liver disease or those at risk for it should consider transitioning from consuming mostly refined grains to whole grains (42). According to research by Peng et al., (40) increased hepatic fat accumulation and IR contribute significantly to the association between meat consumption and the onset of NAFLD. Additionally, red meat was linked positively to serum ferritin, which might raise the risk of NAFLD. It is expected that the above nutrients in the form of a dietary pattern will have synergistic effects on each other to reduce the risk of NAFLD effectively.

Our findings indicate a very high fiber intake, a relatively optimal dietary fat profile, and a pretty low sugar intake for cases and controls, unseen in western countries. Because in the nutritional culture of Iran, unlike western countries, foods are mainly based on grains and bread, especially whole grain bread and vegetables, and fried foods are used less; it is not far from expected that the intake of fiber is high and the intake of fats, refined grains and sugar is low.

Our study had several strengths. It was the first case-control study investigating NAFLD's nutrient pattern and risk. In addition, trained dieticians collected dietary intakes in face-to-face interviews to reduce measurement bias. There are some limitations to this study. In case-control studies, there is an inevitable selection and recall bias. The data were analyzed using factor analysis to identify patterns. Research decisions significantly affect the number of derived factors and factor loadings of nutrients in each pattern. Underreporting alcohol consumption in Iranian society due to cultural-religious issues is one of the limitations that can affect the differentiation between alcoholic and non-alcoholic fatty liver disease; however, because the city of Isfahan has a mainly religious population and the fact that the data used in this study were from a few years ago when alcohol consumption was still low in Iranian society. Therefore, it can be said that the underreporting cases in the present study are not high enough to impact the study results significantly. Finally, despite using a validated semi-quantitative FFQ, measurement errors may occur due to dietary recall.

## Conclusion

This study showed that high compliance to a nutrient pattern characterized by fructose, vitamin C, vitamin A, pyridoxine, and potassium mostly supplied from fruits, vegetables, and nuts is inversely proportional to the odds of NAFLD. Our study found no significant association between other patterns and the odds of NAFLD. Also our findings indicate a very high fiber intake, a relatively optimal dietary fat profile, and a pretty low sugar intake for cases and controls, unseen in western countries. However, these initial findings need to be approved with further studies to confirm the relationship between nutrient patterns and NAFLD.

## Data availability statement

The raw data supporting the conclusions of this article will be made available by the authors, without undue reservation.

## Ethics statement

The studies involving human participants were reviewed and approved by Ethics Committee of Isfahan University of Medical Sciences (Ethic code: 395408). The patients/participants provided their written informed consent to participate in this study.

## Author contributions

AH, AS-s, and FT designed the protocol of the study. HA, EM, and MA conducted the study. FT analyzed the data and drafted the manuscript. AS-s and SS interpreted data and critically revised the manuscript. All authors read and approved the final manuscript.

## Funding

This study is related to project NO. 1399/64282 financially supported by Student Research Committee, Shahid Beheshti University of Medical Sciences, Tehran, Iran.

## Conflict of interest

The authors declare that the research was conducted in the absence of any commercial or financial relationships that could be construed as a potential conflict of interest.

## Publisher's note

All claims expressed in this article are solely those of the authors and do not necessarily represent those of their affiliated organizations, or those of the publisher, the editors and the reviewers. Any product that may be evaluated in this article, or claim that may be made by its manufacturer, is not guaranteed or endorsed by the publisher.

## Abbreviations

NAFLD: Non-alcoholic fatty liver disease
NPs: nutrients patterns
FFQ: Food frequency questionnaire
MET: Metabolic equivalent
BMI: Body mass index
IR: insulin resistance
DMT2: type 2 diabetes mellitus
MetS: metabolic syndrome.
